# Morphological changes in intraretinal microvascular abnormalities after anti-VEGF therapy visualized on optical coherence tomography angiography

**DOI:** 10.1186/s40662-020-00195-2

**Published:** 2020-06-01

**Authors:** Osama A. Sorour, Nihaal Mehta, Caroline R. Baumal, Akihiro Ishibazawa, Keke Liu, Eleni K. Konstantinou, Sarah Martin, Phillip Braun, A. Yasin Alibhai, Malvika Arya, Andre J. Witkin, Jay S. Duker, Nadia K. Waheed

**Affiliations:** 1 Department of Ophthalmology, New England Eye Center, Tufts Medical Center, 800 Washington Street, Box 450, Boston, MA 02111 USA; 2 Department of Ophthalmology, Tanta University, Tanta, Egypt; 3 The Warren Alpert Medical School of Brown University, Providence, Rhode Island USA; 4 Department of Ophthalmology, Asahikawa Medical University, Hokkaido, Japan; 5 University of Hawai’i John A. Burns School of Medicine, Honolulu, HI USA; 6 Yale School of Medicine, New Haven, Connecticut USA

**Keywords:** Retina, Ischemia, Anti-VEGF, DME, PDR, OCTA

## Abstract

**Background:**

To examine the baseline morphological characteristics and alterations in intraretinal microvascular abnormalities (IRMAs) in response to anti-vascular endothelial growth factor (anti-VEGF) treatment, documented by optical coherence tomography angiography (OCTA) in diabetic eyes.

**Methods:**

In this retrospective study, IRMAs were evaluated with multimodal imaging (fundus photography, fluorescein angiography, OCTA) in treatment-naïve diabetic eyes before and after anti-VEGF treatment for diabetic macular edema (DME) and/or proliferative diabetic retinopathy (PDR) and compared to diabetic control eyes with similar diabetic retinopathy (DR) severity that did not receive anti-VEGF therapy. The morphological characteristics of IRMAs on enface OCTA imaging were graded by masked readers at baseline, then after anti-VEGF therapy in treated eyes or after observation in control eyes. Characterization of interval changes in an IRMA were based on the following parameters: branching, vessel caliber and area of adjacent capillary non-perfusion.

**Results:**

The treated group included 45 IRMA foci from 15 eyes of 11 patients, while the control group included 27 IRMA foci from 15 eyes of 14 patients. Following anti-VEGF treatment, enface OCTA demonstrated that 14 foci of IRMA (31%) demonstrated regression with normalization of appearance of the capillary bed, 20 IRMAs (44%) remained unchanged, six IRMAs (13%) progressed with enlargement or development of new IRMAs and five IRMAs (11%) demonstrated complete obliteration defined as IRMA disappearance with advancing capillary drop-out. In the control group, 17 IRMA (63%) remained stable, 8 IRMAs (29.6%) progressed and 2 experienced total obliteration (7.4%). The difference in rank order between the two groups was statistically significant (*p* = 0.022).

**Conclusions:**

In eyes with DR status post anti-VEGF therapy, foci of IRMAs have a variable course demonstrating one of four possible outcomes: regression, stability, progression or complete obliteration. In contrast, none of the untreated control diabetic eyes demonstrated regression of IRMAs, consistent with known progression of DR severity in high risk eyes. Morphologic evaluation of IRMAs with OCTA may help to monitor changes in retinal blood flow as well as the response to anti-VEGF treatment.

## Background

Diabetic retinopathy (DR) is a common complication of diabetes and is a major contributor to vision impairment globally. The worldwide prevalence of DR is rapidly increasing, and is expected to cause visual deterioration in more than 6 million patients in the US by 2030 [1]. Intraretinal microvascular abnormalities (IRMAs) are a common finding in the eyes of diabetics with severe non-proliferative (NPDR) and proliferative diabetic retinopathy (PDR). The modified Airlie House Classification provided the clinical terminology of IRMA, but did not establish whether this vascular abnormality represented new intraretinal blood vessel formation or dilation of pre-existing blood vessels [2]. The Early Treatment of Diabetic Retinopathy Study (ETDRS) provided standard photographs 8A and 8B for IRMAs, which was defined as tortuous intraretinal vascular segments located in standard fields 3–7, with caliber ranging from barely visible to 31 μm based on color stereoscopic photograph evaluation [3, 4].

Intravitreal injection of an anti-VEGF agent is the primary treatment for diabetic macular edema (DME) and may now be used to treat severe NPDR and PDR [5]. However, data regarding the morphological characteristics of IRMAs and changes in response to anti-VEGF treatment are limited [6]. While fluorescein angiography (FA) has been the gold standard imaging modality for imaging the retinal vasculature in DR, it is invasive with potential adverse side effects, limiting its repeatability in closely monitoring treatment response and retinal vasculature changes over time. In addition, FA is two-dimensional and dye leakage may obscure vascular details. Moreover, its resolution at the level of the capillary bed is low. Structural OCT does not allow for visualization of blood flow and cannot directly visualize newly formed vessels. Thus, comprehensive morphologic evaluation of IRMAs by these two modalities is restricted [7]. Optical coherence tomography angiography (OCTA) is a non-invasive, depth-resolved imaging modality that can visualize the retinal microvasculature in great detail, and has been shown to be useful in DR to examine neovascularization, capillary non-perfusion, and IRMAs with higher resolution compared to conventional FA [8].

As development of IRMAs has been associated with worsening severity of DR and hypothesized to be secondary to progressive ischemia [9], a detailed study of IRMA morphology and treatment response may be of clinical importance. The purpose of this study is to evaluate the short-term morphologic characteristics of treatment-naïve IRMAs before and after anti-VEGF treatment compared to control DR eyes using OCTA.

## Methods

This retrospective study was conducted at the New England Eye Center of Tufts Medical Center (Boston, MA, USA). The study protocol was approved by the Institutional Review Board at Tufts Medical Center. The research adhered to the Declaration of Helsinki and the Health Insurance Portability and Accountability Act of 1996 [10].

Study population included treatment-naïve diabetic patients who received anti-VEGF injections for DME and/or PDR at New England Eye Center between January 2016 and December 2019, and whose medical records, included OCTA imaging pre- and post-anti-VEGF injection. A control group of diabetic patients with severe NPDR or PDR who had not received anti-VEGF treatment, and for whom consecutive OCTA imaging was obtained within 3 to 12 months of follow-up, were enrolled for comparison. In addition to already mentioned criteria, patients to be included in both study and control groups should be 18 years or older and had signed a consent to use their data. To avoid bias in selecting the control group, ~ 850 diabetic patients in the NEEC records were randomly selected, we identified those patients that fulfill in/exclusion criteria, and finally included all remining patients who demonstrated the presence of IRMAs in their OCTA images, with inclusion of all present IRMAs in these OCTA scans. We have included patients that received one or consecutive (not more than 12 weeks apart) multiple injections with either anti-VEGF drugs: aflibercept, ranibizumab, or bevacizumab. Exclusion criteria were previous treatment with panretinal laser photocoagulation (PRP) or anti-VEGF injection. Additionally, patients with choroidal neovascularization (CNV), uveitis, uncontrolled glaucoma, endophthalmitis, vitreomacular traction, prior intravitreal corticosteroid injection or previous vitrectomy or any ocular surgery within the last 6 months were excluded. Eyes with significant media opacity limiting the quality of OCTA images were also excluded. Inclusion was allowed for control eyes with previous focal laser treatment for DME that was performed more than 1 year prior to enrollment. Data collected included baseline demographics (age, gender), duration of diabetes, medical history, the type and time of any administered ocular medication or intervention, and current ophthalmologic examination findings, including best-corrected visual acuity, slit-lamp biomicroscopy, dilated fundus examination, and central retinal subfield thickness (CST) using Cirrus OCT (Carl Zeiss Meditec, Inc., Dublin, California, USA).

OCTA 6 × 6 mm images were obtained using the Optovue RTVue-XR Avanti SD-OCT (Optovue, Inc., Fremont, CA, USA) and the Carl Zeiss Cirrus 5000 HD-OCT (Carl Zeiss Meditec, Inc., Dublin, California, USA). In addition, 12 × 12 mm OCTA images from the Carl Zeiss PLEX Elite 9000 (Carl Zeiss Meditec, Inc., Dublin, California, USA) with good quality and high signal-noise ratio were also included. Pre- and post-anti-VEGF treatment imaging of each patient needed to have been performed on the same machine to include the patient in the study.

The enface total retinal OCTA slab was used for reviewing IRMAs. This slab is automatically generated from the Carl Zeiss machines and was manually created in the Avanti system by selecting an inner boundary at the internal limiting membrane (ILM) and an outer boundary set at 40 μm above the retinal pigment epithelium (RPE) to avoid any flow signals from choriocapillaris. OCTAs were reviewed simultaneously with FA and color fundus photographs (CFP) to identify foci of IRMAs by a retina specialist (O.S.). Grading of retinopathy severity was done according to the International Clinical Diabetic Retinopathy Severity Scale [11]. IRMAs were defined as dilated, tortuous intraretinal vascular loops that were not consistent with the natural distribution of normal capillaries, with intraflow that does not breach the ILM on the OCTA B-scan.

Two masked, trained readers (KL, SM) independently performed the morphological evaluation of predetermined IRMAs in all OCTA images, and the interval changes following anti-VEGF treatment (treatment group) or observation (control group). Any discrepancy was resolved by open discussion. Graders gave IRMAs an overall grading score using a scale from 1 to 4 (Table 1), based on the following IRMAs characteristics: branching of the IRMA lesion, caliber of IRMAs and capillary non-perfusion area around the IRMAs lesion.

**Table 1:** Grading scheme for IRMA morphology

Grading score	Description
4 Regression of IRMA	IRMA branches become incorporated into the surrounding capillary bed and convoluted IRMA branches assume a more normal branching pattern. This improvement may be partial (Fig. 1a,e) or nearly total (Fig. 1c, g) and is associated with improvement in perfusion of surrounding area.
3 Stable IRMA	IRMA remains relatively unchanged, with the same number of branches. Vessel caliber and surrounding area of non-perfusion remain constant (Fig. 2e, Fk).
2 Progression of IRMA	Increase in IRMA vascular branching (Fig. 2b, h), and/or appearance of newly formed IRMA (Fig. 2a, g). Associated with enlargement of the surrounding region of non-perfusion with loss of previously visualized capillary bed.
1 Obliteration of IRMA	Disappearance of IRMA with progression of capillary non-perfusion. End-stage progression with nearly total or complete disappearance of IRMA (IRMA drop-out) (Fig. 2c, i) and advancement of surrounding capillary drop-out.

These individual parameters (branching, caliber, surrounding area of non-perfusion) were graded using a 3-point scale (increase = 3, no change = 2, decrease = 1). In addition, qualitative grading of the change in ischemia in the non-perfusion area around the IRMAs lesion was performed by examining the appearance or disappearance of capillaries in the surrounding area.

Statistical analysis was performed using SPSS v.25 (SPSS, Inc., Chicago, IL, USA). The Chi-squared test was used to compare DR stage between the treated and control groups The Mann-Whitney U test was used for comparison of the overall grade between the treatment and control group. The weighted Kappa was used to judge the agreement between graders for all scores. Spearmen’s rank-order correlation was done to assess correlation between grading parameters. The Kruskal-Wallis test was done to assess the difference in IRMAs response to treatment between baseline morphological patterns. *P*-values less than 0.05 were considered to be significant.

## Results

The anti-VEGF treated group included 45 areas of IRMAs in 15 eyes from 11 patients, and the control group included 27 areas of IRMAs from 15 eyes of 14 patients. In the treated group, there were 7 males (63.7%) and 4 females (36.3%) with a mean age of 54 years (range: 27–75 years). The control group included 8 males (57.2%) and 6 females (42.8%) with a mean age of 56 years (range: 41–90 years). In the treated group, 9 eyes had PDR (60.0%) and 6 eyes (40.0%) had severe NPDR. In the control group, 8 eyes had PDR (53.3%) and 7 eyes had severe NPDR (46.7%). There was no significant difference in the DR stage between the treated and control groups (X^2^ = 1.6, *p* = 0.2). However, eyes in the treated group had either DME (*n* = 12) or active high-risk retinal neovascularization – based on the ETDRS criteria (*n* = 3) [12, 13] as the major indication for anti-VEGF injection. Inversely, eyes in the control group did not have these clinical features requiring immediate therapy, according to ETDRS guidelines.

Treated eyes received an average of 3.2 (range: 1–8) consecutive intravitreal anti-VEGF injections. Total number of injections throughout the study was 46 injections, comprising 17, 4, and 25 injections by aflibercept, ranibizumab, and bevacizumab, respectively. Pre-treatment OCTA images were obtained between 0 and 12 days before injection (median 0 days), while post-injection OCTAs were obtained 25 to 70 days after final injection (median 35 days). The mean length of follow-up in the control group was 217 days (range: 94–350 days).

**Fig. 1:**
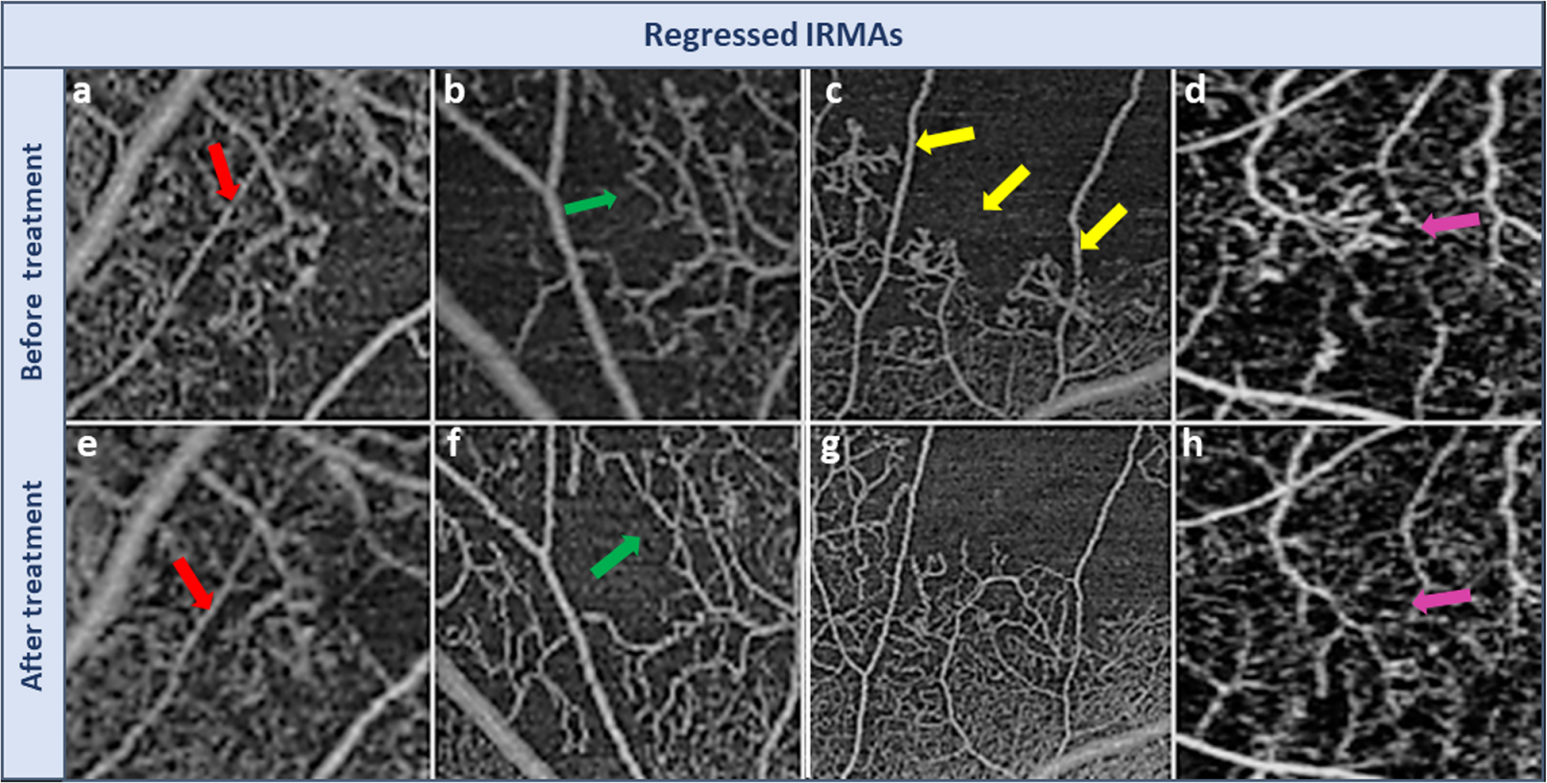
Improvement in IRMA with anti-VEGF treatment (Regressed IRMAs). The upper row demonstrates foci of IRMAs (arrows) prior to intervention and the lower row demonstrates improvement of the same IRMA foci after anti-VEGF injection. Note that IRMA branches become incorporated into the surrounding capillary bed with a decrease in convolution of vessels to form a more normal branching pattern. There is also a reduction in the surrounding adjacent area of non-perfusion. Improvement may be partial (red and green arrows) or very marked (yellow and purple arrows)

**Fig. 2:**
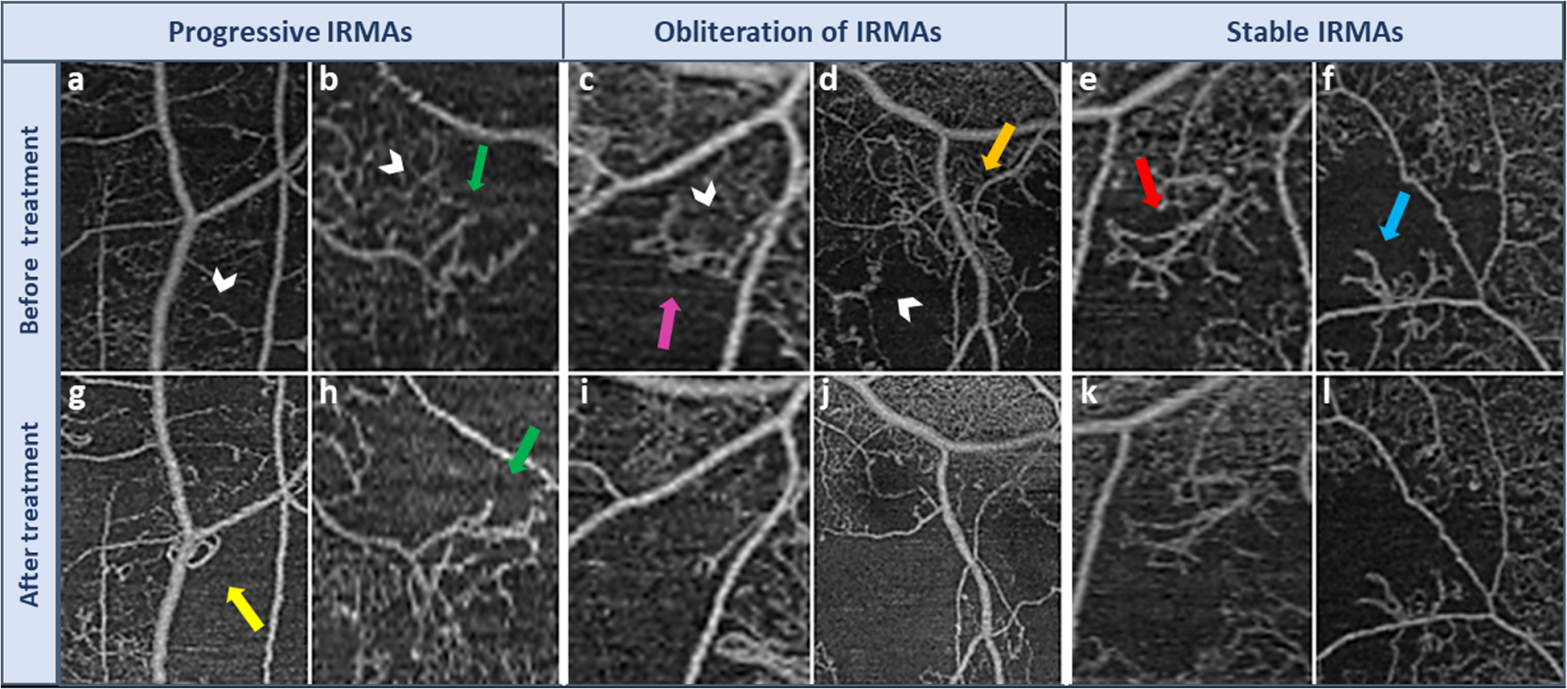
Worsening and stability of IRMAs after anti-VEGF treatment. The upper row demonstrates foci of IRMAs (arrows) prior to intervention and the lower row demonstrates the same IRMA foci after anti-VEGF injection. Panels (**a**, **b**, **g** & **h**) represent progression of IRMAs (yellow and green arrows). The surrounding area of non-perfusion has enlarged with loss of previously adjacent capillaries (white arrow heads). IRMAs in this category either developed more branching (green arrows) or a newly formed IRMAs appeared (yellow arrow). Panels (**c**, **d**, **i** & **j**) represent IRMA obliteration (drop-out), which is an end-stage progression of ischemia leading to massive obliteration of the vascular bed (white arrow heads), which eventually included the IRMAs itself (purple and orange arrows). Panels **e**, **f**, **k** & **l** demonstrate stable IRMAs (red and light blue arrows) where there is no change in the area of non-perfusion, IRMAs caliber, or branching

**Fig. 3:**
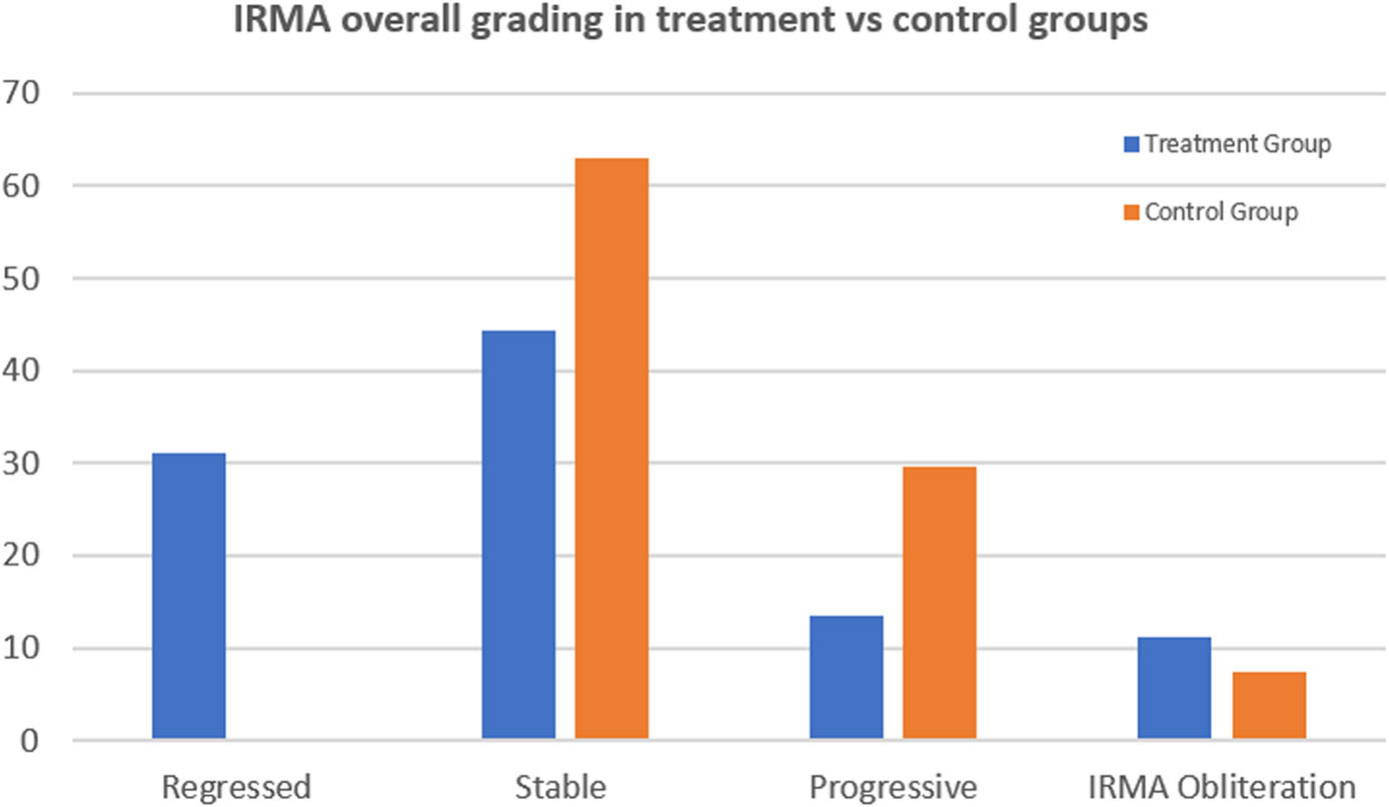
A graph representing difference of change in IRMAs foci between anti-VEGF-treated and control eyes. Note regressed IRMAs were only observed in the treatment group

Assessment of the change in IRMAs morphology after anti-VEGF treatment demonstrated regression of 14 foci of IRMAs (31%). Amongst this group, regression of IRMAs was nearly complete in 10 IRMAs (22%) and partial in 4 IRMAs (9%). 20 foci of IRMAs (44%) remained stable, 6 IRMAs (13%) progressed and 5 foci of IRMAs demonstrated complete obliteration of the IRMAs as well as capillary non-perfusion (11%). In the control group, there were 0 regressed IRMAs, 17 stable IRMAs (63%), 8 progressive IRMAs (29.6%), and 2 that disappeared (7.4%). The difference between the two groups was statistically significant (*p* = 0 .022; Figs. 1, 2 and 3).

With respect to the individual grading parameters, the number of branches decreased in 12 IRMAs (26.7%), remained stable in 21 (46.7%), and increased in 12 (26.6%) after treatment (Fig. 2h). The vessel caliber decreased in 15 IRMAs (38.5%) (Fig. 1g & h), remained stable in 20 (51.3%), and increased in 4 (10.3%) following treatment. The area of surrounding capillary non-perfusion decreased in 11 IRMAs (24.4%) (Fig. 1f, h & i), remained stable in 24 (53.3%) and increased in 10 (22.3%) (Fig. 2g, h, i & j). In the control group, the number of branches decreased in 2 IRMAs (11.1%), was stable in 11 (61.1%), and increased in 5 (27.8%). The vessel caliber decreased in 1 IRMA (3.7%), was stable in 25 (92.5%), and increased in 1 (3.7%). The area of capillary non-perfusion increased in 7 lesions (25.9%), remained stable in 20 (74.1%), and did not decrease in any. Analysis of the correlation between parameters of grading (branching, caliber, perfusion,) with each other in the anti-VEGF treatment group, revealed a significant negative correlation between perfusion and caliber (*P* < 0.0001).

**Fig. 4:**
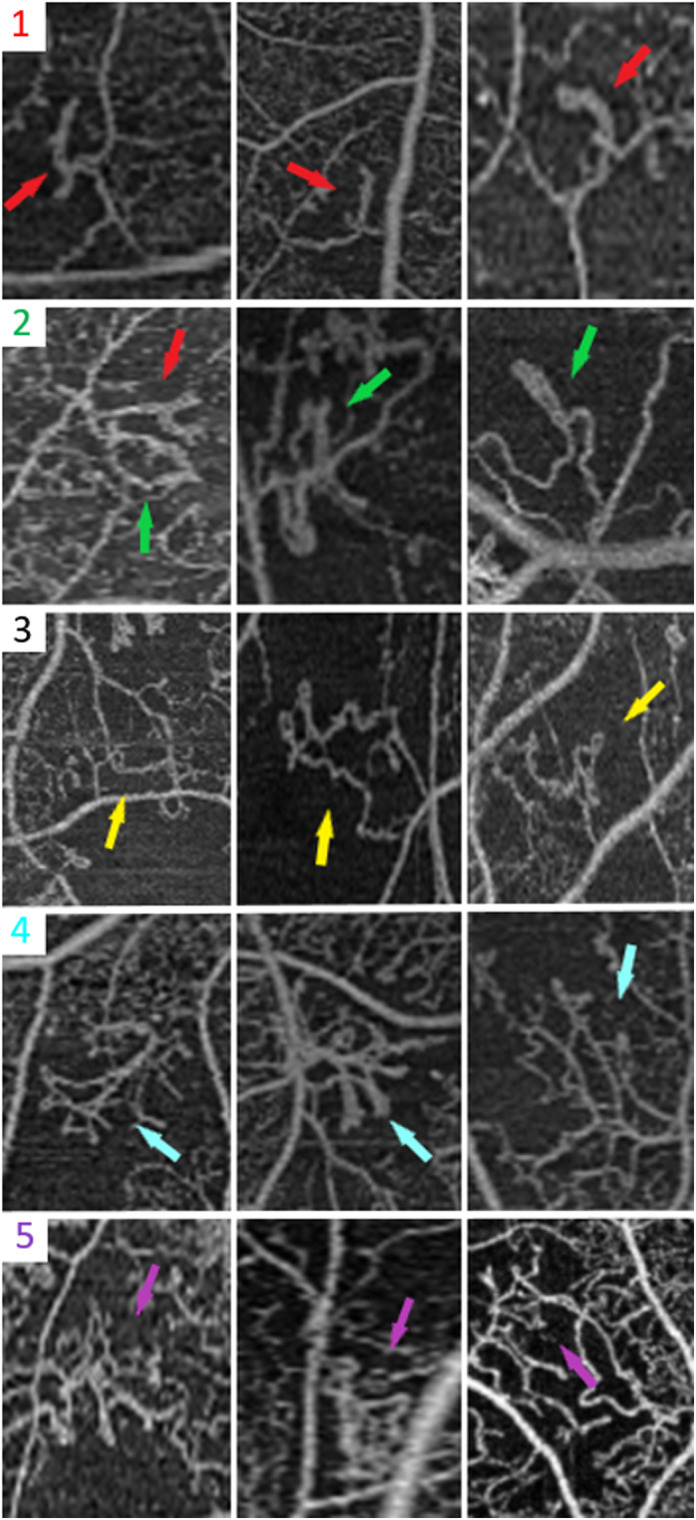
Baseline morphological appearances of IRMAs. Row 1: Dilated trunk IRMA (red arrow) has increased caliber compared to the surrounding capillary bed and end blindly, with either a straight or a slightly curved shape. Row 2: Looped IRMA (green arrow) is a circular loop vascular channel originating from and draining into the same vessel. Row 3: Twisted loop or pigtail IRMA (yellow arrow) is a loop with self-rolling to resemble single or adjacent figures-of-eight with a more irregular twisting pigtail appearance. Row 4: Sea-fan shaped IRMA (blue arrow) has a branching pattern of vascular growth and the feeding and draining vessels are confined to a narrow base, forming the outline of a triangle. Row 5: Net-shaped IRMA (purple arrow) has a complex shape such that the feeding and draining vessels are not confined to a narrow base and cannot be accurately identified, and this IRMA has a rectangular or irregular outline

**Fig. 5:**
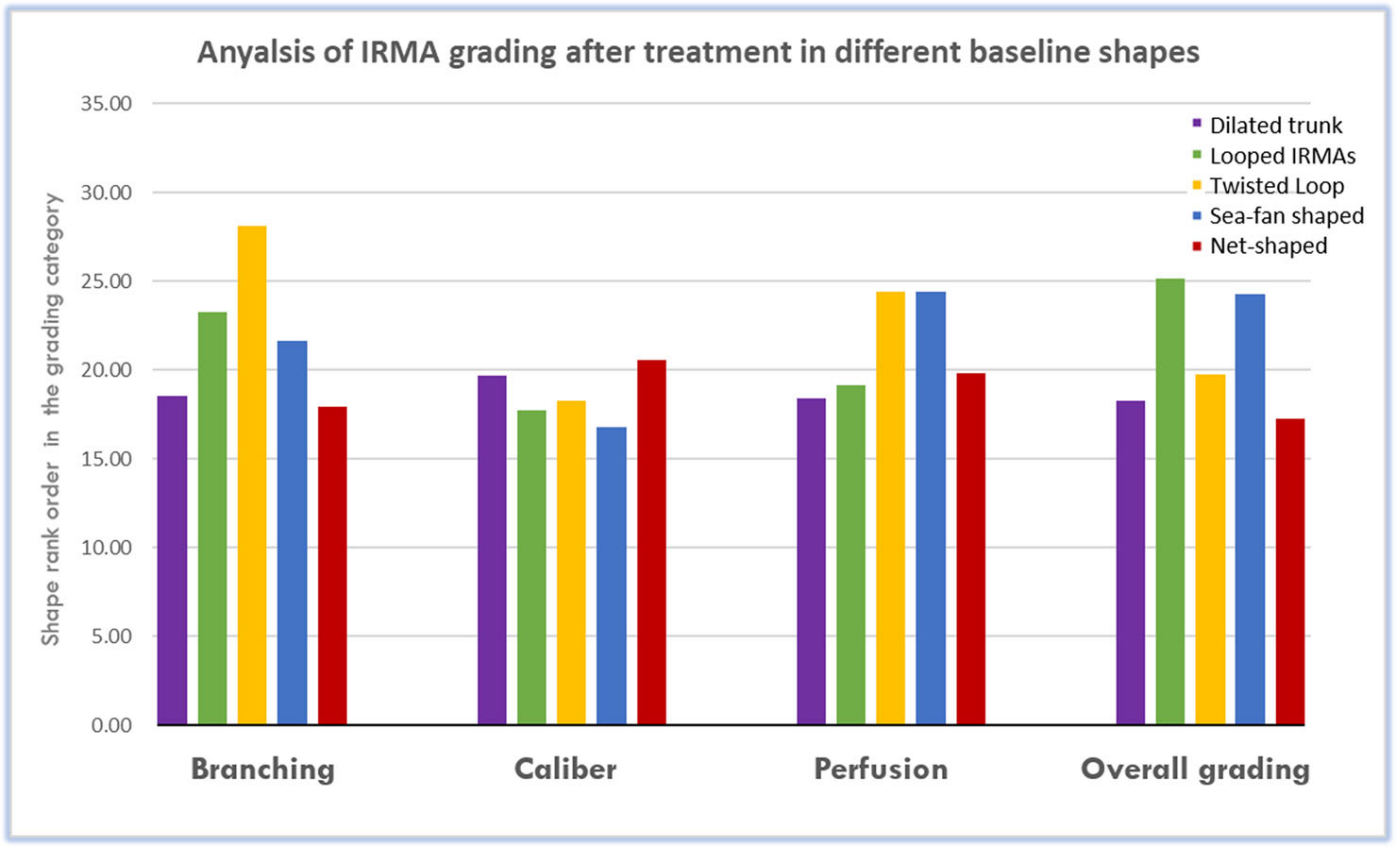
Diffrence in IRMAs changes in after anti-VEGF treatment across various baseline morphological shapes. There was no significant difference in the grading, across all parameters, pre- and post-treatment between IRMAs morphologies (all *p* > 0.4), suggesting no clinical prognostic significance to the different morophologies

Qualitatively, IRMAs were of various morphological appearance, including dilated trunk, loop, pigtail, sea-fan shaped, and net-shaped (Fig. 4). There was no difference noted in IRMA response to anti-VEGF treatment between the different morphologies (Fig. 5).

Mean CST in the whole treatment group, and in the DME subgroup (excluding eyes with PDR and no DME that received anti-VEGF for neovascularization), were 356.9 ± 123.7 μm (range: 227–647 μm) and 399 ± 122.4 μm before treatment, respectively. CST then improved to 300.7 ± 57.7 μm (range: 232–460 μm) and to 314.8 ± 62.3 μm after the final injection (*P* = 0.64 & 0.013, respectively). No significant correlation was found between the change in CST and the grading of IRMA after anti-VEGF treatment (*P* = 0.23).

There was no association between the number of anti-VEGF injections and change in IRMAs; some foci of IRMA showed regression and improvement of ischemia after one injection, while other IRMAs progressed, and ischemia increased despite multiple injections. It is notable that none of the regions of IRMA progressed to be neovascularization elsewhere (NVE) in the treatment group or control group within the follow up duration.

## Discussion

OCTA is a useful tool to identify and monitor IRMAs. Previous studies indicated that IRMAs may be more readily detected with OCTA compared to colored fundus photographs [14]. Previous OCTA studies characterized IRMAs as abnormal, branching, dilated retinal vessels that do not protrude above the ILM and are associated with areas of capillary drop-out [14, 15] with a similar appearance on FA [16]. However, a detailed evaluation of precise IRMAs morphology using OCTA and interval evaluation after anti-VEGF treatment has not previously been conducted.

Our morphological grading, including the overall score of IRMAs after anti-VEGF treatment was based on three parameters: branching of IRMA, caliber of vessels, and surrounding area of capillary non-perfusion. These parameters were chosen in accordance with histopathological findings that emphasized caliber enlargement as a feature of IRMA compared to normal capillaries [17], as well as growth of IRMAs at regions of capillary network obliteration [18].

Although few reports demonstrated progression of macular ischemia with anti-VEGF treatment of DME [19, 20], a randomized multicenter study concluded that anti-VEGF treatment was not inferior to PRP in the treatment of PDR, with a documented slowing and even improvement of DR after anti-VEGF treatment [5, 21, 22]. Capillary revascularization with PRP treatment has long been documented, highlighting the possibility of reversibility of DR following treatment, even in advanced cases [23]. However, these FA-based studies did not investigate changes that occurred in pre-existing IRMAs. Also, there are no FA-based studies that investigate morphological changes in IRMAs with anti-VEGF treatment [24].

Our study found that some patients after anti-VEGF injections demonstrated noticeable improvement in ischemia, evidenced by reperfusion of previous areas of capillary drop-out, while others remained stable or showed progression of vascular loss. IRMAs which were present at the edge of the non-perfusion areas underwent parallel morphological changes. Some IRMAs improved nearly completely or partially (regressed IRMAs) with changes in appearance to more closely resemble the normal capillary network (Fig. 1). These changes included decrease in caliber, incorporation of IRMAs branches into the surrounding capillaries, and straightening of complex convolutions to resemble a more normal branching pattern. Our results of different degrees of improvement in regressed IRMAs are comparable to previous FA studies that have described two patterns of reperfusion in the retinal avascular area after PRP: recanalization, which has a vascular pattern similar to normal retinal vasculature, and intraretinal neovascularization, which presented with different vascular pattern [25]. In our study, progression of IRMAs because of increase in ischemia manifested with either appearance of new IRMAs or an increased number of larger caliber vessels (Progressive IRMAs) (Fig. 2). End-stage ischemia with wide obliteration of the vascular bed also resulted in IRMAs obliteration or drop-out (Fig. 2). Although in our study the control group did not show improvement in any IRMAs lesions, there have been previous reports of spontaneous reperfusion without treatment [26].

This study also recognized various baseline shapes of IRMAs s, including dilated trunk, loop, pigtail, sea-fan shaped, and net-shaped (Fig. 4). Although we generally noticed that eyes with a more advanced stage of DR and a higher degree of retinal ischemia tended to have more complex forms of IRMAs, further quantitative research would be needed to confirm this. Early histopathologic studies described various shapes of these abnormal vascular channels, of what would also come to be termed IRMAs, including Y-shaped and S-shaped forms [18]. In our small analysis, the shape of IRMAs was not a predictor of treatment response to anti-VEGF (Fig. 5). Further studies should examine if the shape of IRMAs is correlated with response to different forms of treatment, such as PRP, and thus whether it has clinical significance.

Overall, our findings are consistent with previous FA-based research showing improved perfusion in some cases after anti-VEGF treatment [22]. However, we also documented the changes in IRMAs after anti-VEGF treatment. We have introduced a more comprehensive scheme for evaluation, where the changes that occur in the surrounding area of non-perfusion are graded separately from the changes in the IRMAs. However, this study was a qualitative assessment and further studies that quantify ischemia and correlate it to changes in IRMAs would be valuable.

The present study has several limitations. First, it was retrospective and so subject to the limitations of that study type. There was also a relatively small sample size. In addition, the treatment group was given anti-VEGF injections for the indication of DME and active high-risk retinal neovascularization—these conditions were not present in the control group, which may indicate more severe disease in the treatment group. However, the treatment group showed improvement in 31% of IRMAs, which may suggest that degree of improvement could be even higher if the two groups had exactly the same DR severity [27]. Additionally, the time of follow-up in the control group was longer than that of the treatment group. However, our goal was to allow for sufficient time for any spontaneous improvement to occur, and we limited our follow-up time in the treatment group to prevent documenting changes that may not have been attributable to anti-VEGF treatment. Other limitations include the slight change in focus and resolution between successive OCTA images of some IRMA lesions. Additionally, some eyes in the control group received prior focal laser treatment, but not eyes in the treatment group. However, these eyes received focal laser more than 1 year before enrolment in the study. Our final limitation was the diversity of number and type of anti-VEGF injections.

## Conclusion

Morphological assessment of IRMAs using OCTA may help in the identification of focal changes in perfusion and monitoring of treatment response to intravitreal anti-VEGF injection. Following treatment, IRMAs can improve, remain stable, or progress, or become totally obliterated in advancing ischemia. In untreated eyes, IRMAs appear to remain stable, progress, or undergo complete obliteration. The ability of OCTA to evaluate IRMAs may be important in the context of low sensitivity of global OCTA metrics in monitoring perfusion changes with treatment.

## Data Availability

The datasets used during the current study are available from the corresponding author on reasonable request.

## Abbreviations

Anti-VEGF: Anti vascular endothelial growth factor
DME: Diabetic macular edema
DR: Diabetic retinopathy
IRMA: Intraretinal microvascular abnormality
NPDR: Non-proliferative diabetic retinopathy
OCTA: Optical coherence tomography angiography
PDR: Proliferative diabetic retinopathy
PRP: Panretinal photocoagulation
